# Canine acquired pneumonia caused by *Bordetella bronchiseptica*

**DOI:** 10.1016/j.idcr.2023.e01922

**Published:** 2023-10-31

**Authors:** Luis Lantigua Tatem, Todd Veale, Christopher Richardson, Tracy Luckhardt

**Affiliations:** a The University of Alabama at Birmingham, School of Medicine, Division of Pulmonary, Allergy, & Critical Care Medicine, 1900 University Blvd. Tinsley Harrison Tower, Suite 422, Birmingham, AL 35294, United States; b The University of Alabama at Birmingham, School of Medicine, Department of Medicine, 510 20th Street South, Birmingham, AL 35294, United States; c The University of Alabama at Birmingham, School of Medicine, Division of Pulmonary, Allergy, & Critical Care Medicine, 1900 University Blvd. Tinsley Harrison Tower, Suite 513A, Birmingham, AL 35294, United States

**Keywords:** *Bordetella bronchiseptica*, Dog, HIV, Pneumonia, Zoonosis

## Abstract

Here, we present the case of a 55-year-old male with HIV and persistent lymphopenia who developed a paroxysmal severe cough for over three weeks. Microbiology studies were positive for abundant colonies of *Bordetella bronchiseptica*. He reports that his dog was also ill with a severe cough, suggesting a possible canine-to-human transmission. This zoonosis has been increasingly recognized and possesses significant morbidity and mortality, especially in immunocompromised hosts.

## Highlights

• *Bordetella bronchiseptica* is an increasingly recognized zoonosis.
• A high index of suspicion and assessment for animal exposures are essential in diagnosing this entity.
• Advising immunocompromised patients regarding this disease is of utmost importance.

## Case

A 55-year-old male presented to the pulmonary clinic with a history of episodic productive cough for three weeks. The cough was productive with brown, blood-tinged sputum, paroxysmal episodes severe enough to induce emesis, and mild hemoptysis. He denied fevers, chills, or rigors. He had pleuritic chest pain that worsened during the coughing spells and progressive mild to moderate dyspnea on exertion. His medical history included HIV with persistent lymphopenia despite HAART (lymphocyte CD4 count of 55 cells/cmm, CD4% of 11%), HIV viral load 25 copies/mL, persistent leukopenia, Kaposi’s Sarcoma status post chemotherapy and radiation therapy. His medications included emtricitabine 200 mg daily, tenofovir alafenamide 25 mg daily, etravirine 400 mg, raltegravir 1200 mg daily, sulfamethoxazole-trimethoprim 800 mg /160 mg daily. His family history was positive for diabetes mellitus in his mother and heart disease in his brother. On physical exam, he had a temperature of 97.9 F, heart rate of 86 bpm, oxygen saturation of 94% on room air, and blood pressure of 113/79 mm Hg; he was alert and in no distress, the skin was normal, respirations were non-labored, and was not actively wheezing. The heart exam had no murmurs, and he had no lower extremity edema.

The patient reports living at home with his dog, who had been coughing multiple times before his illness. The dog had also recently received the “kennel cough” vaccine a few months prior. He reported close contact with the dog, including sharing the bed with his pet.

**Fig. 1:**
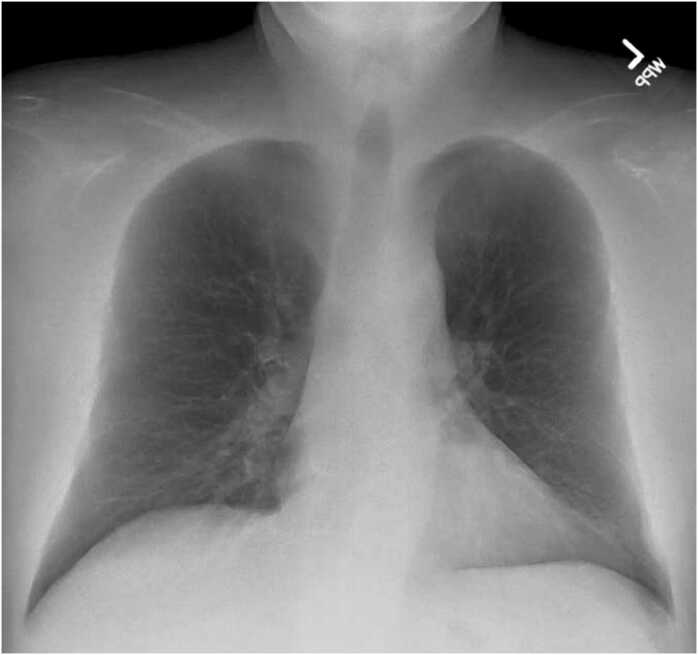
Chest X-ray: posteroanterior view. Mild bronchial wall thickening.

Due to concerns about pneumonia, a chest x-ray was ordered (Fig. 1), showing mild bronchial wall thickening—no focal airspace opacity. A sputum culture was obtained, initially showing Gram-negative bacilli, later identified as abundant colonies of *Bordetella bronchiseptica* on MacConkey agar medium. MicroScan WalkAway 96 plus susceptibility testing showed resistance to cefepime (MIC >16) and trimethoprim/sulfamethoxazole (MIC > 2), intermediate to ceftazidime (MIC >16), sensitive to ciprofloxacin (MIC ≤ 1), levofloxacin (MIC ≤0,5), meropenem (MIC ≤1), piperacillin/tazobactam (MIC ≤16). He was prescribed levofloxacin 750 mg daily for ten days and reported significant improvement in his coughing spells.

## Discussion

*Bordetella bronchiseptica* was first isolated and identified in 1911 in dogs, and it was named Bacillus bronchianis [1]. Bacillus bronchicanis was chosen since the organism was isolated from the respiratory tracts of dogs suffering from distemper [2]. It is a Gram-negative, obligate aerobic coccobacillus known to cause disease in domesticated animals [3]. This bacteria is recognized as one of the most common pathogens for canine infectious respiratory disease complex (CIRDC) [4]. Human cases of *Bordetella bronchiseptica* are rare, most common in patients with immunodeficiencies and abnormal lung parenchyma; other presentations such as meningitis [3], and bloodstream infections [5] have been described (Table 1).

**Table 1:** Clinical presentation of *Bordetella bronchiseptica* in humans.

No. of patients	Clinical presentation	Ref.
1	A 61-year-old man with renal transplant and bacteremia.	Powers et al. [4]
1	A 15-year-old M with fever, forceful coughing, and hemoptysis.	Woods et al. [13]
1	A 61-year-old male with HIV/AIDS with fevers, shortness of breath, and intermittent chest pain.	Gujju et al. [6]
1	A 42-year-old man with HIV/AIDS with pneumonia, fevers, and hypotension.	Galeziok et al. [14]
1	A 52-year-old with lung cancer with persistent productive cough.	Monti et al. [15]
1	A 77-year-old male with ulcerative colitis on infliximab with traumatic cerebrospinal fluid leak complicated by meningitis.	Radcliffe et al. [3]
22^a^	5/22 (23%) had an acute exacerbation of chronic obstructive pulmonary disease (COPD); 5/22 (24%) had an acute respiratory infection; 3/ 22 (14%), had a fever; 2/22 (9%) had pneumonia; 2/22 (9%), chronic cough; 1/22 (5%), like syndrome; 1/22 (5%), respiratory distress; 1/22 (5%), acute bronchospasm; and 1/22 (5%), laryngotracheitis plus otitis media.	García-de-la-Fuente et al. [1]
1	42-year-old female with alcoholic liver disease with pneumonia and peritonitis.	Dlamini et al. [16]
1	56-yr-old male with continuous ambulatory peritoneal dialysis and relapsing peritonitis.	Won et al. [17]
1	A 71-year-old male with bacteremia and pancreatic abscess.	Matic et al. [18]
1	A 7-year-old child with cystic fibrosis with persistent and recurrent pneumonia.	Khatib et al. [19]
1	A 7-year-old child status post bone marrow transplant and pneumonia.	Choy et al. [12]
1	A 35-year-old quadriplegic male with acute hypoxic respiratory failure	Clements et al. [20]
1	A 61-year-old female status post kidney–pancreas transplant with pneumonia after exposure to recently vaccinated dogs.	Gisel et al. [9]

a A total of 11 (50%) patients were female, and the median age was 60 years (range, <1–90). Nineteen patients were admitted to the hospital, and 4 patients (18%) required admission to the intensive care unit. Four patients had repetitive episodes and were the only ones who had documented exposure to dogs or cats.

In a case series of 30 patients of PLWHA, animal exposure history was given for 73% of cases, with 55% reporting animal contact [6]. Between 2016 and 2022, the percentage of U.S. households who own dogs increased by 6.1% points, from 38.4% to 44.5%, while the percentage of households that own cats increased by four percentage points, from 25% to 29% [7]. Evidence of exposure to *Bordetella bronchiseptica* is frequently found in healthy and diseased dogs, and client-owned dogs are as likely to be infected as kenneled dogs [4]. In an extensive survey of pathogens associated with respiratory disease in multi-cat (5 cats) households in nine European countries, *Bordetella bronchiseptica* was detected by PCR in 5% of cats from households with the disease and 1.3% without the disease [8]. As more Americans are exposed to animals as pets, recognizing and becoming familiarized with this disease is of utmost importance, especially in populations with a higher risk of significant disease as advanced age, immunocompromised, and chronic parenchymal lung disease such as cystic fibrosis. Another concern in the immunocompromised host is the postulation of possible transmission by animals that recently received a live-attenuated, intranasal *Bordetella bronchiseptica* vaccination [9].

Antimicrobial susceptibility of *Bordetella bronchiseptica* suggests high resistance rates to beta-lactams, cephalosporins, macrolides, and trimethoprim/sulfamethoxazole but low MICs to tetracyclines and quinolones [10], [11]. The evidence on treatment is based on animal studies, case reports, and in vitro susceptibility data; treatment duration ranges from 7 to 14 days with monotherapy with doxycycline or levofloxacin; others suggest combination therapy [12].

In conclusion, *Bordetella bronchiseptica* is an increasingly recognized pathogen with heterogeneous clinical presentations, most commonly presenting with persistent cough and respiratory symptoms but also with isolated bacteremia, meningitis, intrabdominal abscess, and peritonitis. Most cases suggest contact with domesticated animals, suggesting this entity is a zoonosis of clinical importance. A high index of suspicion and a good anamnesis for risk factors is vital in patients with a compatible clinical presentation, especially in patients with immunocompromising conditions.

## Funding

This research received no specific grant from public, commercial, or not-for-profit funding agencies.

## Ethical approval

Ethics committee approved.

## Consent

Written informed consent was obtained from the patient for publication of this case report and accompanying images.

## Declaration of Competing Interest

No conflict of interest.
